# Light therapy for bipolar disorders: Clinical recommendations from the international society for bipolar disorders (ISBD) Chronobiology and Chronotherapy Task Force

**DOI:** 10.1080/19585969.2025.2533806

**Published:** 2025-07-24

**Authors:** Pierre A. Geoffroy, Laura Palagini, Tone E. G. Henriksen, Patrice Bourgin, Corrado Garbazza, Claude Gronfier, Yuichi Esaki, Diego C. Fernandez, Raymond W. Lam, Heon-Jeong Lee, Michel Lejoyeux, Julia Maruani, Klaus Martiny, Greg Murray, Rixt F. Riemersma-Van Der Lek, Philipp Ritter, Peter F.J. Schulte, Daniel J. Smith, Michael Terman, Jamie M. Zeitzer, Dorothy K. Sit

**Affiliations:** a Département de psychiatrie et d’addictologie, AP-HP, GHU Paris Nord, DMU Neurosciences, Hôpital Bichat – Claude Bernard, Paris, France; b NeuroDiderot, Inserm, FHU I2-D2, Université Paris Cité, Paris, Franc; c Centre ChronoS, GHU Paris – Psychiatrie & Neurosciences, Paris, France; d Institute for Cellular and Integrative Neurosciences, CNRS UPR 3212 & Strasbourg University, Strasbourg, France; e Department of Neuroscience, Psychiatric Section, University of Pisa, Azienda Ospedaliera Universitaria Pisana (AUOP), Pisa, Italy; f Division of Mental Health Care, Valen Hospital, Department of Research and Innovation, Fonna Health Trust, Haugesund, Norway; g CIRCSom (International Research Center for ChronoSomnology) & Sleep Disorders Center, Strasbourg University Hospital, Strasbourg, France; h Centre for Chronobiology, University Psychiatric Clinics (UPK), Basel, Switzerland; i Lyon Neuroscience Research Center (CRNL), Inserm U1028, CNRS UMR5292, Université Lyon 1, Bron, France; j Department of Psychiatry, Fujita Health University School of Medicine, Aichi, Japan; k Division of Pediatric Ophthalmology, Cincinnati Children’s Hospital Medical Center. Department of Ophthalmology, College of Medicine, University of Cincinnati, Cincinnati, USA; l Department of Psychiatry, University of British Columbia, Vancouver, Canada; m Department of Psychiatry, Korea University College of Medicine, Seoul, Republic of Korea; Chronobiology Institute, Korea University, Seoul, Republic of Korea; n Mental Health Centre Copenhagen, Mental Health Services of the Capital Region of Denmark and Department of Clinical Medicine, University of Copenhagen, Copenhagen, Denmark; o Centre for Mental Health and Brain Sciences, Swinburne University, Melbourne, Australia; p Mental Health Services Drenthe, Assen, the Netherlands; q Department of Psychiatry and Psychotherapy. Faculty of Medicine and University Hospital Carl Gustav Carus, TUD Dresden University of Technology, Dresden, Germany; r Mental Health Service Noord-Holland-Noord, Alkmaar, Netherlands; s Division of Psychiatry, Centre for Clinical Brain Sciences, University of Edinburgh, Edinburgh, Scotland, UK; t Center For Environmental Therapeutics, Worcester, MA, USA; u Columbia University Psychiatry, New York, NY, USA; v Department of Psychiatry and Behavioral Sciences, Stanford University, Stanford, CA, USA; w Feinberg School of Medicine, Department of Psychiatry and Behavioral Sciences, Northwestern University, Chicago, IL, USA

**Keywords:** Bipolar disorder, depression, seasonal affective disorder, light therapy, phototherapy, chronotherapy

## Abstract

The International Society for Bipolar Disorders (ISBD) Chronobiology and Chronotherapy Task Force conducted a comprehensive review to deliver concise evidence-based recommendations on the use of bright light therapy (BLT) for bipolar disorder (BD). Adjunctive BLT is likely an efficacious acute treatment for bipolar depression as implicated by higher quality evidence. The position of maintenance BLT for relapse prevention awaits further investigation. Protocols of effective BLT in BD are similar to parameters indicated for treatment of seasonal and non-seasonal major depressive disorder. Anti-manic prophylaxis (especially for BD-I) and clinical monitoring are recommended with initiation of and ongoing light treatment. Administer BLT daily, preferably in the morning or at mid-day. If mornings are prohibitive, then mid-day exposure, implemented to avoid excessively early wake times, is an acceptable alternative. Informed by the literature, target 30 min/day of BLT exposure. Patients wary of emergent hypomania or partial responders, can initiate 15 min/day and increase by 15 min each week to full response (or 30–60 min/day by the fourth week). Consider patient centred outcome assessments to evaluate mood response, safety and side effects. Clinical improvement is typically observed within 1–2 weeks, with response/remission expected by 4–6 weeks. Integration of BLT with other chronotherapeutic strategies may enhance long-term efficacy.

## Introduction

People who live with bipolar disorder (BD) spend more time grappling with depressive symptoms than manic symptoms. Bipolar depression can diminish quality of life and lead to unfavourable outcomes, characterised by persistent functional and cognitive impairments, recurrent mood episodes, treatment-refractory illness, high burden of medication side effects and increased suicide risk (Judd et al. 2002, 2003a, 2003b). First line treatments for bipolar depression exhibit limited efficacy, while electroconvulsive therapy (ECT) and wake therapies, although highly effective, are not widely available (Vieta et al. 2010; Grunze et al. 2013; Gottlieb et al. 2019; Wirz-Justice and Benedetti 2020). Antimanic therapy or ‘mood stabilizers’ often falls short in treating depression, prompting the addition of conventional antidepressants. Such combination regimens are promising but may carry risks of affective mood switch, rapid cycling, and suicidality (Vieta et al. 2010; Grunze et al. 2013). Given the challenging therapeutic landscape, development of novel treatments tailored to manage and prevent bipolar depressive episodes is a public health imperative. Despite mounting evidence to support the clinical efficacy of bright light therapy (BLT) and newfound insights into the physiological mechanisms, BLT is largely underutilised and poorly understood by mental health clinicians (Wirz-Justice et al. 2004; Geoffroy et al. 2020). Bridging the gap between research findings and clinical application is essential to fully harness the benefits of BLT in BD.

The historical connection between light and mood dates back to ancient times (Geoffroy 2020). From Wong Tai’s venerable observations linking seasonal light variations to mood fluctuations, to Hippocrates’ advocacy of ‘heliotherapy’ in 400 BC, early scientists recognised the influence of sunlight on human well-being and posited the therapeutic potential of light interventions. Modern uses of BLT are informed by neuroscientific innovations launched in the 1980s and onward. Foundational discoveries and ground-breaking progression of this work are elaborating the putative mechanisms, that encompass the light detection, neural pathways and integrate with knowledge of chronobiology, homeostatic sleep processes, wake systems and monoaminergic neurocircuits to explain the pathophysiology of BD (Harvey 2008; McClung 2013; Geoffroy 2018; Wirz-Justice and Benedetti 2020).

Mood disorders are characterised by specific patterns, clinical features and comorbid conditions that point to manifestations of disturbances in circadian rhythms (delayed or advanced sleep phase disorders), abnormal sleep-wake cycles (hypersomnia in atypical depression), and altered mood traits predicted by infradian cycles (seasonal variation or premenstrual dysphoric disorder) (Harvey 2008; McClung 2011; Geoffroy 2018)). Chronotherapeutics has increasingly applied a versatile set of evidence based behavioral, environmental and pharmacological interventions to regulate sleep-timing, enhance alertness, improve sleep quality, and manage mood episodes. These chronotherapeutic interventions have received consensus recognition from the International Society for Bipolar Disorders (ISBD) (Gottlieb et al. 2019). Higher quality evidence supports adjunctive BLT as an acute treatment for bipolar depression (Gottlieb et al. 2019). Although generally well-tolerated, anti-manic prophylaxis in BD I subtype and clinical monitoring are recommended with initiation of and ongoing light treatment (Gottlieb et al. 2019). The position of maintenance BLT for relapse prevention awaits further investigation. Daily BLT is identified as an outstanding, primary treatment for seasonal depression (American Psychiatric Association, Practice Guideline for the Treatment of Patients With Major Depressive Disorder (American Psychiatric Association 2000); https://psychiatryonline.org/pb/assets/raw/sitewide/practice_guidelines/guidelines/mdd.pdf and (Maruani and Geoffroy 2019)). Protocols of effective BLT in bipolar depression are similar to parameters indicated for seasonal and non-seasonal major depressive disorder (Gottlieb et al. 2019; Geoffroy et al. 2020). In this evidence-based practice guideline, we aim to propose clinical practice recommendations and applications of bright light treatment in the management of BD.

## The circadian system, SCN-dependent pathways and light (Figure 1)

We believe that a better understanding of the physiological and neurobiological mechanisms underlying BLT is critical for increasing its clinical use. Just as physicians expect to understand the pharmacodynamics of a drug before prescribing it, the mechanisms of light must also be integrated into medical knowledge to facilitate adoption and appropriate application (Geoffroy et al. 2020).

The circadian system is conceptualised as a hierarchical structure, with a master central clock pacemaker overseeing the rhythmic functions of peripheral clocks distributed throughout the body. The central circadian clock in humans, is located in the suprachiasmatic nucleus (SCN) of the hypothalamus (Moore and Lenn 1972; Stephan and Zucker 1972). This small, paired structure, consisting of approximately 20 000 neurons (10 000 x2), is located just above the optic chiasm and below the third ventricle. The SCN orchestrates the daily physiological rhythms by generating or synchronising cycles across nearly all bodily functions, through both direct neural connections with other brain centres (e.g., control of pineal melatonin) and indirectly through the release of humoral signals that influence the timing of the clocks found in most tissues (e.g., gut oscillator) (Dibner et al. 2010). Often described as the conductor of an orchestra, the SCN ensures that tissue-specific (peripheral) oscillators remain harmonised, enabling the precise temporal coordination of various physiological processes.

Alignment of the circadian clock in the SCN with the external light/dark cycle is primarily set by input from the retino-hypothalamic tract. This anatomical pathway (that functionally transmits photoreceptor signals from the retina to the SCN) consists of axons originating from a subset of retinal ganglion cells (RGCs) that receive inputs from rod-cone networks, and express the photopigment melanopsin, making them intrinsically photosensitive retinal ganglion cells (ipRGCs) (Baver et al. 2008). As photoreceptors in the retina, ipRGCs detect light through the melanopsin signalling pathway, which is particularly sensitive to blue light with a maximal peak at ∼480 nm. Within the SCN, neurons integrate light signals with non-photic inputs, such as those mediated by serotonin from the medial raphe nucleus (Hay-Schmidt et al. 2003), neuropeptide Y signals from the intergeniculate leaflet nucleus of the thalamus (Janik and Mrosovsky 1994), and melatonin from the pineal gland (Shibata et al. 1989). These photic and non-photic signals, collectively known as ‘zeitgebers’ (from the German ‘time giver’), help synchronise internal rhythms with the external environment. Under natural conditions, the detection and processing of regular zeitgebers leads to robust entrainment or synchronisation of the internal time-keeping mechanisms. However, when these time cues become irregular or desynchronised, the circadian clock’s timing can become erratic, and the amplitude of its oscillation may be reduced (Jewett et al. 1994). A weakened circadian amplitude can, in turn, lead to less effective synchronisation of downstream oscillators, disrupting physiological and behavioural rhythms (Souêtre et al. 1989; Dijk et al. 2012; Harfmann et al. 2017). Output signals from the SCN influence multiple brain circuits, orchestrating a wide range of physiological and behavioural processes. Melatonin production in the pineal gland is one of the best studied pathways of the SCN. In humans, endogenous melatonin circulates at low levels throughout the day. Melatonin production rises exponentially in the evening a few hours before habitual bedtime to a near square wave amplitude (or skewed cosine, (Van Someren and Nagtegaal 2007)) and ceases rapidly within one hour of habitual waketime. Basal levels of melatonin provide feedback onto SCN receptors and influence the timing of its own production, though this effect is much more pronounced during the daytime when circulating melatonin concentrations are typically quite low, as compared to the evening effect (Lewy et al. 1992). Likewise, exogenous melatonin can impact and possibly change the SCN rhythm when administered during the daytime (Lewy et al. 1998). The increased levels of melatonin at night has also been implicated as mechanistically associated with decreases in alertness (Cajochen et al. 2000). In addition to the timing of melatonin being under direct control of the SCN, exposure to light at night can also acutely suppress the production of melatonin in a spectral (Brainard et al. 2001; Najjar et al. 2024) and an intensity-dependent manner (Zeitzer et al. 2000; Prayag et al. 2019) (for review on the effects of light on melatonin and sleep, see: (Prayag et al. 2019)).

The proper timing of sleep emerges from a dual process that balances signals of the SCN circadian pacemaker with sleep homeostasis (Borbély 1982; Dijk and Czeisler 1994). Mediated by health, stress, social and cultural factors, the preferred time of sleep or more broadly, the subjective preference for sleep and wake is defined as ‘chronotype’ (Van Someren and Nagtegaal 2007). Individuals are ‘morning, intermediate, or evening’ types according to their sleep time preference. Homeostatic sleep is an appetitive process such that the more sleep one gets, the less sleep is needed and, conversely, the longer one stays awake, the more sleep is needed. The accumulation and dissipation of homeostatic sleep follows an exponential function (Franken et al. 1991). Operating in tandem with sleep homeostasis is the circadian clock, which is independent of sleep need. The SCN generates a ‘paradoxical’ signal for wake in the hours just before habitual bedtime, and a signal for sleep in the hours just before habitual waketime. While awake, the increased homeostatic drive for sleep is offset by the increased circadian drive for wake. During sleep, the decreased homeostatic drive for sleep is offset by the increased circadian drive for sleep. Together, wake and sleep are consolidated into single daily periods in humans by integrating signals from the SCN, sleep homeostat (Dijk and Czeisler 1995), and possibly from sustained direct light effects (mediated through the melanopsin pathway) (Hubbard et al. 2021).

## SCN-independent pathways for the antidepressant effects of light (Figure 1)

Light treatment is a modality of chronotherapy, traditionally understood as operating through a circadian mechanism whereby light acts as a zeitgeber influencing the SCN (Gottlieb et al. 2019). However, with advancements in mapping the neural projections of ipRGCs in mammals (particularly studies in rodents), emerging evidence suggests that the antidepressant effects of light may be mediated through multiple brain centres, either in parallel with or bypassing the SCN (Maruani and Geoffroy 2022). One compelling example is the recent identification of the perihabenular nucleus (PHb) in the dorsal thalamus of the mouse brain, which has been shown to play a key role in mood regulation (Fernandez et al. 2018; Huang et al. 2019). PHb neurons are particularly responsive to changes in lighting conditions, such as transitions from darkness to light. Light signals processed by the PHb are subsequently routed to regions involved in mood regulation, such as the ventromedial prefrontal cortex, nucleus accumbens, and dorsal striatum (Fernandez et al. 2018).

Human studies using functional MRI have demonstrated the light-intensity–dependent activation in the prefrontal cortex (Sabbah et al. 2022). This effect is consistent with the intensity-dependent suppression of melatonin in response to light (Prayag et al. 2019). Preceding fMRI studies showed acute temporal changes in blood perfusion from blue-light exposure, with an immediate activation of the hypothalamic region and habenular region, followed by activation of the brain stem and monoaminergic brain stem nuclei, specifically the raphe nucleus (serotonin) and locus coeruleus (noradrenaline) (Vandewalle et al. 2009), and neuronal activation of the general cerebrum within 30 min of blue-light exposure. Intriguingly, the patterns of temporal and spatial spread of activity across the inter-connected regions, map onto the neural circuits of mood disorders (Price and Drevets 2010) and correspond with the onset and timing of antidepressant responses to BLT. Together, these findings point to a conserved retino-thalamic-frontocortical pathway in mammals that mediates the effects of light on neural pathways of mood and behaviours among patients with BD.

We must contend with a disproportionate lack of human data from light studies. Promising theories and investigations on light are often piloted in non-human models. Even so, novel findings from such basic science and pre-clinical studies are critical steps in the scientific process that serve to uncover the mechanisms underpinning the role of light in mood and behaviour, and to support the corresponding evidence from clinical studies on the clinical and functional outcomes of BLT in patients with BD.

## Brief, narrative review of efficacy studies of BLT

The literature base on utilising adjunctive BLT (addition of BLT to an existing antimanic agent or ‘mood stabilizer’) for treatment of bipolar depression has expanded immensely across the years (Gottlieb et al. 2019; Lam et al. 2020). Evidence from recent higher quality clinical trials suggest that adjunctive BLT is effective in reducing bipolar depressive symptoms and improving global functioning without much risk for a manic switch (Takeshima et al. 2020). Furthermore, BLT may enhance other dimensions of BD, such as sleep quality, cognitive performance, and anxiety (Sit et al. 2018; Takeshima et al. 2020; Bisdounis et al. 2022). The ISBD Task Force on Chronobiology and Chronotherapy and others have made recommendations to utilise BLT for adjunctive treatment of acute bipolar depression (Gottlieb et al. 2019; Lam et al. 2020). These groups also declared the need for further research in consideration of the study limitations. Of note, the varying light treatment parameters, small sample sizes, brief durations of therapy and inconsistent quality across trials are possible short-comings that could limit generalisability (Gottlieb et al. 2019; Lam et al. 2020).

In a comparison of depression outcomes of light therapy, antidepressant drugs, and combined treatment (BLT with antidepressant)(Geoffroy et al. 2019) meta-analyses showed a clear superiority of combination therapy (standardised mean difference [SMD] = 0.56; *p* < 0.001) and no significant difference in treatment effect of BLT or drug in major depressive episodes (SMD = 0.19; *p* = 0.17) (Geoffroy et al. 2019). Utilising BLT or combination therapy are likely acceptable first-line treatments for depressed patients with a diagnosis of BD or MDD regardless of seasonality (Geoffroy et al. 2019). Lam et al. conducted a high quality meta-analysis of RCTs specific to bipolar depression and showed significant improvements in clinician-rated depressive symptoms (Hamilton Depression Rating Scale, HDRS; (SMD = 0.43; *p* = 0.03) and a clinical response in favour of BLT (odds ratio = 2.32; *p* = 0.024) (Lam et al. 2020). Similar results were found in subsequent meta-analyses (Franken et al. 1991; Dijk and Czeisler 1995; Brainard et al. 2001). Therefore, BLT emerges as an acceptable adjunctive treatment option for management of seasonal and non-seasonal bipolar depressive episodes.

## How to use/implement BLT

To optimise the therapeutic benefits of BLT a comprehensive strategy is crucial (Figure 2). Delivery of BLT is ideally approached following a proper diagnostic evaluation, and complete assessment of the patient’s 24-hour exposure to light and darkness, sources of illumination, habitual sleep/wake times, exercise, sleep hygiene practices (Esaki et al. 2019; 2021a) and schedule constraints (school, work and social obligations) (Ballard et al. 2024). For patients with BD I, antimanic therapy is indicated during BLT and at least 2–4 weeks prior to initiating BLT (Pacchiarotti et al. 2013), to prevent (hypo)manic episodes and to avoid mood induction effects. In contrast for BD II patients, the need for mood stabiliser coverage may depend on the clinician’s discretion (Bahji et al. 2020). Broad consensus supports that treatment guidance and ongoing mood monitoring are provided by a trained clinician (Yatham et al. 2018).

**Figure 1.**
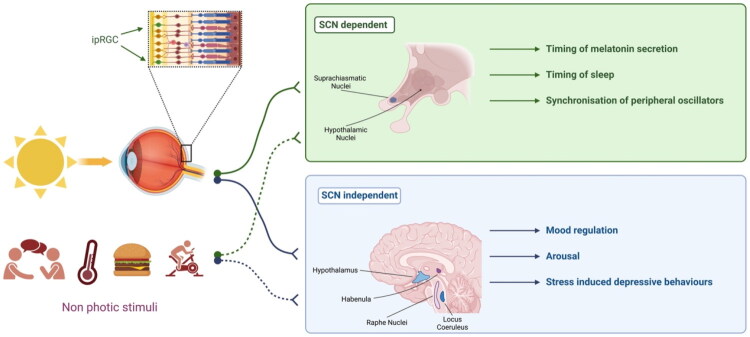
Pathways of bright light therapy (Courtesy of P. Ritter).

**Figure 2.**
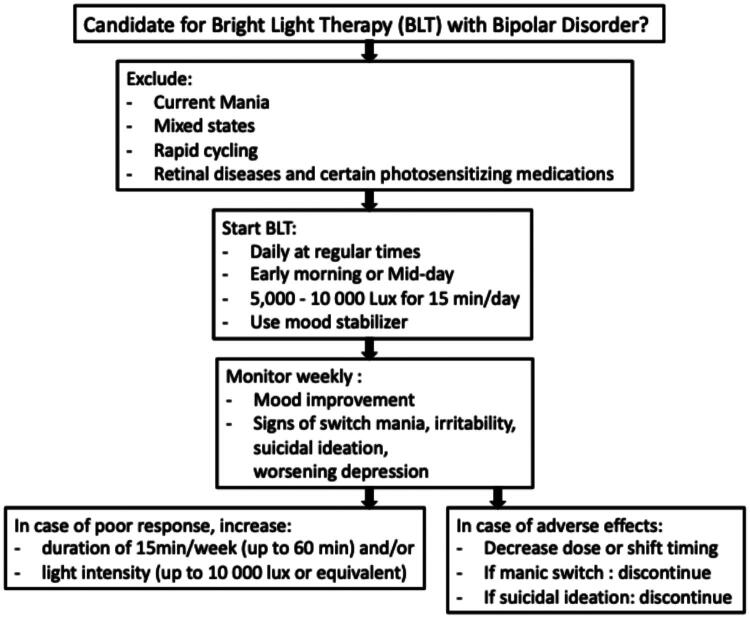
Clinical algorithm: Bright light therapy in bipolar disorder.

### The overall light environment of the patient

Evaluate the individual’s daily exposure to natural and artificial light. Outdoor exposure to natural sunlight in conjunction with daily BLT is advised, given the positive evidence that shows natural light improves mood, reduces depressive symptoms in BD and amplifies circadian rhythms (Esaki et al. 2019; 2021a; 2021b). Spending time outdoors, especially during morning or daylight hours, can enhance the efficacy of BLT and improve general physical health. Outdoor activities may also impart beneficial preventive effects, given the positive associations between increased daytime light exposure and decreased rates of relapse into depressive bipolar episodes (Esaki et al. 2021b).

### Setting the stage for success

Successful utilisation of BLT demands active engagement and persistent motivation on both the parts of the patient and the clinician. Explore feasible ways to integrate the light box sessions into your patients’ daily routine. Define activities which they can enjoy doing, while facing their light box. Clinic patients have shared some of the following comments: During my sessions of light, I can ‘read the newspaper, finish a book chapter, write emails, knit or crochet, do homework, catch up on bills, ride my stationary bike, apply make-up, listen to music, play my flute, journal, perform open-eyed forms of meditation (e.g., mindfulness or breathing-focused exercises)., etc.’ Pay close attention to adherence; daily sessions of light are critical for a robust response. Explain the rationale for BLT’s benefits and potential side effects. In addition to this paper, excellent publications (Chronotherapeutics for Affective Disorders (Wirz-Justice et al. 2013); Lam et al. (Lam et al. 2020)), online resources (cet.org), educational conferences and a vibrant chronotherapy community (www.sltbr.org) are available to support and guide clinicians on implementing light therapy in clinical practice. Help your patients establish a comfortable routine with using their light box (51). Adjustments to the timing and duration of light exposure are dosing refinements that may be applied to augment the patient’s response. Extending a positive rapport, being available to communicate and answer patient questions, scheduling convenient telehealth appointments and building a strong and committed therapeutic alliance are strategies to set the stage for a successful response to BLT (Lam et al. 2020).

BLT is performed indoors using calibrated light boxes, as natural outdoor light can exceed 100,000 lux and is not standardised or controllable for therapeutic use. It should be administered in well-lit rooms to avoid visual discomfort or glare, which can occur when BLT is used in dark environments.

### Contraindications

Acute manic episodes, recent mania or hypomania, mixed symptoms and rapid cycling are contraindications to treatment with BLT. Retinal diseases and certain photosensitising medications (Montgomery and Worswick 2022) such as sulphonamides, chloroquine or St. John’s Wort (Hypericum perforatum), may preclude the use of bright light. Patients with reduced vision, recent changes in vision or past history of eye diseases are advised to have a comprehensive ophthalmic examination prior to treatment (Maruani and Geoffroy 2019). For LASIK surgery patients, the evidence to support the use of BLT in this patient group is limited. But given the reassuring safety profile and urgency to manage worsening symptoms, adjunctive BLT is likely an acceptable intervention but not sooner than 4 weeks post-surgery. Consult with the ophthalmologist and confirm that the eyes are healthy and free from post-surgical complications, such as dry eye, corneal haze, or light-sensitivity before initiation of BLT.

### Light devices

**Figure 3.**
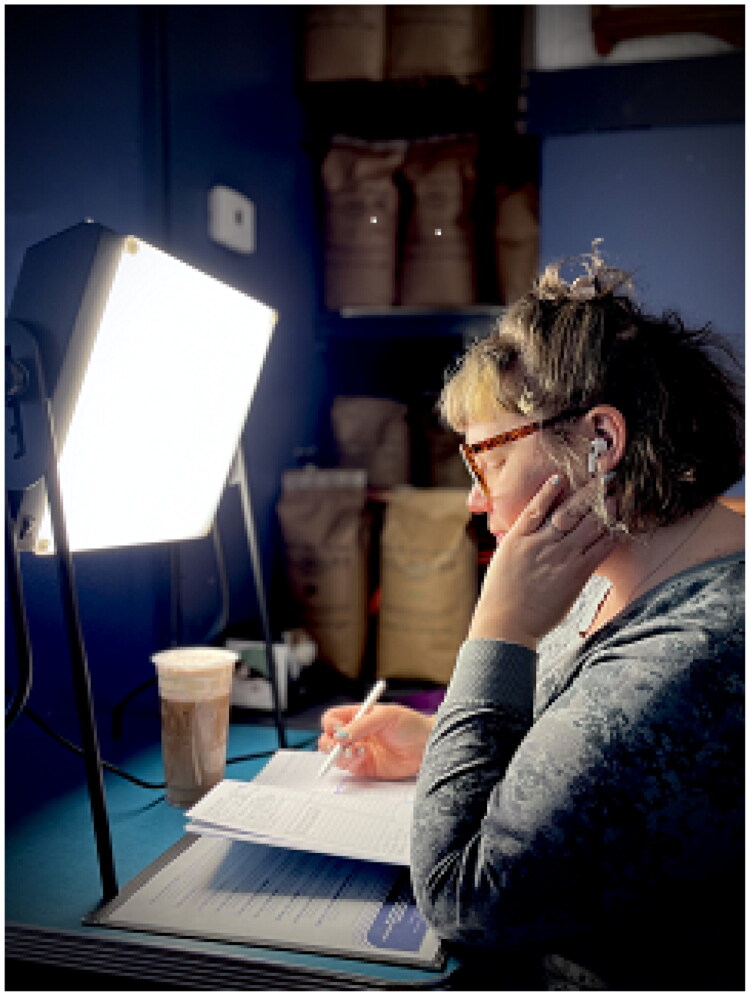
Proper placement of an example of bright light box. (Courtesy of Jessica Rao, www.cet.org).

Light box illumination can vary from fluorescent to light emitting diode (LED) lights; the size of the device can impact usability and effectiveness. The ideal dimensions of a portable unit should measure at least 30 cm (12 inches) in height by 35 cm (14 inches) in length (larger units that exceed the recommended dimensions are acceptable) (Terman and Terman 2005; Lam et al. 2020). Keep in mind that achieving the required intensity of light is dependent upon the proper positioning of the light box. Devices are optimally placed on a desk stand to deliver illumination from above (with a slight downward angular tilt). See Figure 3. Smaller units are not recommended; they can produce excess glare that causes eye discomfort and often requires a patient’s seating much closer to the light source than is comfortable. The unit should be constructed with diffusion screen and emit diffuse, low-glare lighting across a broad visual field. Avoid staring directly at the unit to reduce the risk of eye discomfort. Staying exposed to the BLT lamp without any interruption for 30–60 min daily is difficult for certain patients. If amenable, patients can stand and stretch for a few minutes before resuming the session of light exposure. Innovative treatment lamps are designed and installed in hospital wards or personal space within built environments; these units can expand the entire wall or ceiling surface and permit longer distances of illumination to reach the patient compared to portable lamps at eye level. When planning such installations for traditional (short) session of bright light therapy, it is important to provide sufficient light level.

### Treatment parameters: Basic protocols and directions for acute treatment

The steering parameters of light treatment include intensity, timing of exposure, duration of daily sessions, and spectral composition. The recommended protocols are based on parameters which were established in SAD, with some adjustments for BD to prevent hypomanic or manic switches. Further studies are needed to define the optimal timing and light source parameters of patients who utilised BLT to manage (non-seasonal) bipolar depression.

#### Light intensity

The dose of light (BLT dosage) is a function of light intensity and the duration of daily exposure. In a general inverse relationship, exposure to higher intensity light requires less daily exposure. Lower intensities are effective but require longer exposure; from earlier trials, SAD patients received 2500 lux for 2 h a day, 5000 lux for 1 h a day for instance (Eastman et al. 1998; Terman and Terman 2005; Terman 2007). To manage bipolar depression, select a bright, white, ultraviolet (UV)-filtered light 5,000–10,000 lux at a distance of 30–33 cm (12–13 inches) from the eyes (Gottlieb et al. 2019; Lam et al. 2020). Patients with BD may be sensitive to lower light intensities (< 10 000 lux) so the protocols may vary (Dallaspezia and Benedetti 2020; Wang et al. 2020). The antidepressant effect was achieved even when low (<5000 lux) light intensity was used (Dallaspezia and Benedetti 2020; Wang et al. 2020) although usage parameters are not yet fully standardised (Geoffroy et al. 2018). Generally, light intensities below 1500 lux are regarded not effective in depression.

By convention (and in available RCTs), photopic ‘lux’ is the unit of light intensity that quantifies illuminance or the total power of light that falls on a surface from any direction (Enezi et al. 2011) and depends on both luminance emitted by the light source and the distance of the sensor (such as distance from the light box diffusion screen to the corneal surface of the eyes). Melanopic lux is a measure of illuminance, that weighs the optical power at each wavelength as well as the spectral composition of polychromatic light (Enezi et al. 2011). Because melanopic lux is more physiologically relevant compared to photopic lux, future BLT trials are likely to utilise the melanopic measure.

#### Timing of bright light exposure

The ideal time to administer BLT is in the morning (shortly after waking, between 7:00 AM and 9:00 AM) or at mid-day (between 12:00 PM and 2:30 PM (Sit et al. 2018)). Early administration, around 6:00 AM or 8:00 AM, may allow for a better chance of remission (Lewy et al. 1982; Eastman et al. 1998; Terman and Terman 2005; Terman 2007). Morning light may provide a higher response compared to other times of day (Terman et al. 1998). For non-responders at mid-day, move the timing of light gradually to the morning to improve response (Sit et al. 2007; 2018; Sit and Haigh 2019). Patients with extreme chronotypes or circadian phase shift disorder may benefit from a personalised approach that takes into consideration their habitual late or early bed/wake times, and integrates carefully timed bright light treatment with proper sleep hygiene practices (Rosenthal et al. 1985; Meesters and Houwelingen 1998; LeGates et al. 2014; Chojnacka et al. 2016; Benedetti 2018; Sit et al. 2018; Zhou et al. 2018; Gottlieb et al. 2019).

#### Duration of bright light exposure

Although many of the published studies started with a full-dose of light, recent RCTs innovated the dose titration protocol (Sit et al. 2007; Dauphinais et al. 2012; Geoffroy et al. 2018; Sit et al. 2018). In this approach, the duration of daily exposure is progressively increased each week to build a beneficial mood response and to mitigate risks of unpleasant emergent effects (headaches, eye discomfort and insomnia). Utilising this judicious approach may serve to limit the likelihood of inducing a (hypo)manic switch or rapid cycling, particularly among patients prescribed an early morning exposure. For instance, the protocol could start at 15 min/day and progressively increase by 15 min weekly until target duration of 60 min/day is reached at four weeks, while observing for response and tolerance, especially signs of restlessness, irritability or a switch to hypomania (Geoffroy et al. 2018; Sit et al. 2018; Gottlieb et al. 2019). Hypomanic symptoms that appear during BLT can resolve with a simple decrease in dosage (duration and/or intensity) or by stopping BLT if it is not enough to quickly reduce (hypo)manic symptoms (Sit et al. 2007; Lam et al. 2020; Geoffroy et al. 2025). For some individuals, start with a lower dose of light such as 7.5 min/day (Dauphinais et al. 2012; Geoffroy et al. 2018) and gradually increase exposure time to a target dose of 30–60 min/day, until symptoms are fully remitted, full functioning is restored, or prohibitive side effects preclude further titration. Frequent monitoring and careful dose adjustments by the clinician are essential to support a positive and well-tolerated response and to promptly manage mood destabilisation. Utilise patient centred outcome assessments to evaluate mood severity, safety including suicidal risk (https://www.cssrs.columbia.edu, (Posner et al. 2011)), and side effects (Ballard et al. 2024) and to inform decisions on individual dosing needs. The self-reported, 9-item Patient Health Questionnaire (PHQ-9, (Kroenke et al. 2001)), 7-item generalised anxiety disorder scale (GAD-7, (Spitzer et al. 2006)) and the clinician rating instruments, such as the Structured Interview Guide for the Hamilton Depression Rating Scale with Atypical Depression Supplement (SIGH-ADS) (Williams and Terman 2003) and Young Mania Rating Scale (Young et al. 1978), are validated tools which can effectively track symptom severity and treatment response in clinical and research settings. In hospital daily assessments are more feasible and would support the rapid adjustment of more intensive light therapy protocols than in outpatient settings.

#### Light spectrum considerations

The optimal spectra of light utilised for treatment of bipolar depression is less well investigated but white light (fluorescent (Chojnacka et al. 2016; Yorguner Kupeli et al. 2018) and light emitting diode (LED) (Zhou et al. 2018)) have been most widely used and appear to be the most effective. Low-intensity blue light, while effective in shifting circadian rhythms, has yet to be demonstrated as having efficacy for depressive disorders (Do et al. 2022). Ultraviolet wavelengths are not required for antidepressant efficacy and, because of the potential for long-term harm, only UV filtered light devices are recommended for clinical purposes.

### Time course of effects for acute BLT

The time course of effects for BLT can vary. Some patients may notice improvements within a few days, while others may take several weeks (Rosenthal et al. 1985; Meesters and Houwelingen 1998; LeGates et al. 2014; Chojnacka et al. 2016; Benedetti 2018; Sit et al. 2018; Zhou et al. 2018; Gottlieb et al. 2019). Symptomatic remission should be expected by four to six weeks of treatment (Sit et al. 2018; Lam et al. 2020). BLT generally produces faster antidepressant benefits than pharmaceutical antidepressants and also helps regulate circadian rhythm alterations that may be present in patients (Geoffroy et al. 2019). Regular follow-ups are necessary to assess progress and make any needed adjustments to the treatment plan.

### Side effects and safety

In general, BLT has shown an excellent level of tolerance among patients. Adverse effects such as headaches, dizziness, sleep disturbances, eye strain, nausea, symptoms of excessive activation (agitation, anxiety, arousal) or (hypo)manic switch are usually transient and mild (Terman and Terman 2005; Benedetti 2018; Gottlieb et al. 2019; Lam et al. 2020). Of note, in the LuBi trial (Geoffroy et al. 2025), the only one recent randomised controlled trial that has specifically examined hypomanic switch rates in a dose-escalation design, 2 out of 34 participants (6%) experienced a hypomanic switch. One patient with BD type II switched after 45 min of morning light exposure in week 1, and another with BD type I switched after 45 min of mid-day exposure in week 4. Additionally, five patients (15%) exhibited subsyndromic hypomanic symptoms – three in the morning group (all exposed to 45 min: two BD type II and one BD type I) and two in the mid-day group (30 min, both BD type II). Overall, 20.6% of participants (7/34) exhibited either subsyndromic or full-blown hypomania. All symptoms resolved within 3 days after dose reduction. These observations, though limited by sample size, found no significant associations between manic symptoms and the week of treatment, the starting dose, the cumulative dose, or the morning versus mid-day exposure. These results suggest that, within a structured and carefully monitored framework, dose-escalated BLT can induce manageable side effects while providing therapeutic benefits. Both morning and mid-day administrations were generally well tolerated up to 45 min of exposure.

No ocular disorders related to BLT were observed in a meta-analysis assessing short-term ocular effects, nor in a five-year follow-up study (Gallin et al. 1995; Brouwer et al. 2017). Suicidal ideation and mood induction are uncommon outcomes of BLT (Sit and Haigh 2019; Lam et al. 2020). Notably, the data show a good safety profiles and lower manic switch rates than with pharmaceutical antidepressants (Dallaspezia and Benedetti 2020). The side effects are mild and diminish over time. Prior to treatment and with dose titration, inform patients of potential side effects and advise patients to report any severe or persistent treatment emergent effects to their prescribing clinician. Establish regular monitoring and follow up appointments that are enhanced with self-reported or clinician-administered measures to assess patient-centred outcomes and to identify early warning signs of mood worsening or recurrence (Young et al. 1978; Sit et al. 2018; Sit and Haigh 2019; Pulido et al. 2025). During light treatment, mood destabilisation or suicidal ideation requires prompt attention. Advise such patients with worsening depression or increasing suicidal thoughts to reduce the light dose or stop using the light box and to call and speak with the clinician who is monitoring their response. If urgent or in a crisis, then seek immediate medical attention at the nearest urgent care clinic, hospital crisis unit or emergency room. Providing clear, patient recommendations on when to seek help is essential to ensure patient safety and maintain therapeutic efficacy. Although the efficacy of BLT is sometimes attributed to the blue wavelength, this assertion has not been tested properly. The superiority of blue light boxes compared to bright white light for treatment of bipolar depression has not been established (Gallin et al. 1995; Brouwer et al. 2017). In addition, ANSES has conducted a number of expert assessments to better understand the effects of LEDs and blue light on health and to provide recommendations for public authorities to protect the public; its conclusions can be found here: https://www.anses.fr/en/content/leds-blue-light.

### Modifications for seasonal patterns

Modifications for BLT with seasonal episodes include starting treatment in the autumn with first onset of symptoms and continuing through the winter months. First response may appear with BLT in SAD as soon as 3 or 4 days and remission of symptoms is often achieved after 4 weeks of treatment (Wirz-Justice et al. 2013; Gottlieb et al. 2019; Geoffroy and Palagini 2021). Even though the treatment seems effective at 4 weeks, it is recommended to continue during the whole at-risk period (e.g., until spring or summer returns) because many patients can relapse in winter if the treatment is stopped (Eastman et al. 1998; Terman and Terman 2005; Rosenthal et al. 1984; Terman 2007). Finally, BLT may also be used preventively for patients with regularly-occurring episodes of SAD by initiating treatment in September, or at least 2–4 weeks before the expected onset of the depressive period each year, and continuing throughout the at-risk season (Rohan et al. 2016; Garbazza et al. 2022).

### Continuation of treatment

Continued improvement can be observed after six weeks of treatment (see dose-response relationship; (Sit et al. 2018)). However, to date, there are no longer-term follow-up RCTs of BLT in BD (Gottlieb et al. 2019). Given this context, expert consensus recommends that BLT should be continued until full episode recovery and maintained for 1 year to help maintain the benefits achieved during the acute phase and prevent relapse. Develop a personalised maintenance plan based on the patient’s individual needs and response to initial treatment. For maintenance therapy, many patients may be able to reduce the intensity or duration of BLT sessions. Patients with BD who discontinue antidepressant treatment one year after a successful response to a mood stabiliser-antidepressant combination regimen have been shown to have twice the risk of relapse compared to those who continue the combined treatment (70% vs. 36%) (Altshuler et al. 2003). Antidepressants should always be used in combination with a mood stabiliser and are not recommended as monotherapy due to the risk of mood destabilisation. Given the absence of evidence, certain remitted BLT patients may decide to continue BLT for at least (and possibly beyond) one year as a preventive measure against recurrence but additional studies are needed.

### Lifestyle management and integration of BLT

Sleep disturbance is strongly associated with residual mood symptoms, recurrent depressive episodes, and suicidal ideation in patients with BD (Esaki et al. 2021a). Young to mid-aged outpatients with BD (≤ 44 years old) exhibit reduced sleep efficiency, prolonged sleep-onset latency, and increased wake duration after sleep onset, particularly in association with evening white light exposure (Esaki et al. 2021b). Knowing this, assess the quantity and sources of daily light exposure and implement strategies to reduce evening and night-time light exposure. Advise patients to keep the bedroom as dark as possible. Modifying 24-hour light exposure habits can significantly improve sleep quality, which in turn enhances overall treatment outcomes.

Reduced physical activity (Melo et al. 2016), delayed sleep phase (Takaesu et al. 2018), and later timing of daily activities (Esaki et al. 2021a) are predictors of BD depressive episode relapse (Sit et al. 2007; Sit and Haigh 2019). Conversely, increased daytime light exposure (Esaki et al. 2019) and higher relative amplitude of rest/activity rhythms are protective factors, contributing to reduced depressive symptoms (Esaki et al. 2019) and a decreased risk of mood relapse in BD (Esaki et al. 2021b). Encourage patients to engage in regular physical exercise, spend ample time outdoors during daylight hours, maintain consistent sleep schedules, avoid excess evening bright (or blue) light exposure, and limit excessive delays in bedtime (digital/screen curfew one hour before desired bedtime). These behaviours not only complement BLT but are likely to help maintain the treatment effect and prevent relapse.

Feasibility and real-world implementation of BLT protocols remain key challenges, particularly outside specialised centres. While commercially available light therapy devices vary in cost (typically ranging from €100 to €300), access may be limited by lack of reimbursement in many healthcare systems. Patient adherence can also be suboptimal, especially in outpatient settings, due to the need for daily use and sustained engagement over several weeks. In inpatient settings, structured supervision may improve adherence but is constrained by logistical factors, including space availability and staff training. Device standardisation remains a barrier, with variability in size, lux output, UV filtering, and diffusion quality. These issues are particularly salient in low- and middle-income countries (LMICs), where access to reliable devices and ­electricity, as well as cultural perceptions of non-pharmacological treatments, may limit feasibility. Addressing these barriers through implementation research and the development of low-cost, validated light boxes will be critical for broader dissemination.

### Take home messages

In conclusion, effective treatment with BLT may require some behaviour changes from patients. By adding BLT to pharmacological treatments, we anticipate higher response rates and higher remission than pharmacological treatment alone. However, this requires a more tailored collaborative approach, regular follow-ups, and more hands-on involvement from clinicians. The extra effort needed is justified by expectations of a faster remission from this neurologically harmful and dangerous state of BD. On the positive side, patients often appreciate BLT because it gives them an active role in their treatment, whereas medication is sometimes perceived as more passive. By fostering a supportive and informative environment, clinicians can help patients navigate the use of BLT effectively, ensuring better management of bipolar depression and enhancing overall treatment outcomes.

## Summary of key take-home messages and step-by-step guide for the use of BLT

See Tables 1 and 2.

**Table 1.** Protocol for the use of light therapy for major depressive episode in bipolar disorders.

		Bipolar depression	
**Dose**	**Light intensity** depends on **exposure duration**	**First exposition: incremental increase to minimise the risk of adverse effects** (especially if patients at risk of manic shift): **Initial Week (Week 1):** **Begin with 15 min/day of 5,000–10 000 lux light therapy**. **Incremental Increase (Weeks 2–4):** Gradually increase the daily exposure time by 15 min per week. For example: Week 2: 30 min per day Week 3: 45 min per day Week 4: 45–60 min per day Alternate: 2,500 lx x 3 hrs BID (Rosenthal et al.) 5,000 lx x 60 min/d for 2 weeks. (Zhou et al.). 10,000 lx x 30 min/d for 2 weeks (Chojnacka et al. Yorguner-Kupeli et al.)	
	**Distance and Angle from Light Source. Figure 3.**	**Lamp Position:** slight above **eye level** to ensure optimal exposure. **Distance: 30–33 cm (12–13 inches)** from the light source, as specified by the manufacturer’s recommendations. **Exposure:** Ensure **indirect exposure** while seated, facing the light; avoid staring directly into the light to reduce the risk of eye discomfort or strain. Eyes are kept open. Do not walk around the room during the session. Avoid watching TV or activities that cause positioning at extreme angles away from the unit.	
**Color of the light spectrum**		Polychromatic white light has the highest success in terms of efficacy	
**Time of exposure**		**In case of non-seasonal bipolar depression:** Early in the morning (shortly after waking, between 7:00 and 9:00 AM) Or mid-day (between 12:00 and 2:30 PM), *Especially when history of (hypo)manic episodes* Daily, at regular times Consider the chronotype (which can be assessed using the auto-MEQ available at cet.org)	**In case of seasonal pattern**: Prefer early in the morning (e.g., around 8 AM) Consider the patient’s chronotype, which can be assessed using the auto-MEQ available at cet.org. Note. Early exposure in patients who have severe sleep phase delay, may worsen symptoms. Daily, at regular times
**Onset of effect**		∼1–4 weeks *Remission at 4 – 6 weeks*	
**Treatment duration**		**In case of non-seasonal bipolar depression:** Until the disappearance of depressive symptoms	**In case of seasonal pattern**: Until the resolution or disappearance of depressive symptoms. Continue throughout Autumn/winter seasons. Pause or interrupt during the usual remission period (e.g., spring or summer)
**Prevention**		**In case of non-seasonal bipolar depression:** Certain remitted patients may prefer to continue BLT to prevent recurrence for up to one year	**In case of seasonal pattern**: Treatment by BLT is possible in the autumn or at least 2–4 weeks before the depressive period of the year
**Prevention of a (hypo)manic switch**		By using an adequate dosage of a mood stabiliser with antimanic properties for at least 2–4 weeks before initiation of BLT, especially in patients with BD I	
**Adverse effects**		**(Hypo)Manic episode** Suicidal ideation Worsening depression **Mild side effects**: headache, dizziness, sleep disturbance, eye strain, nausea, activating symptoms (agitation, anxiety, arousal)	
**Contraindications**		Hypersensitivity to light or photophobia Photosensitising drugs In the event of pre-existing eye abnormalities (especially conditions affecting the retina) or photosensitising treatments: light therapy only after an ophthalmological examination In the absence of existing evidence, BLT may generally be performed after LASIK surgery, but not before 4 weeks post-surgery and only following an ophthalmological consultation to confirm that the eyes are healthy and free from post-surgical complications, such as dry eye, corneal haze, or sensitivity to light	

**Table 2.** Step by step guide. implementation of blt for depressed patients with bipolar disorder.

**Key Points**. **Figure 2**.
Maintenance therapy with an anti-manic agent, especially in BD I, is indicated for at least 2–4 weeks before initiation of BLT and during treatment (Yatham et al. 2018). Continuation of stable dosed antidepressant with anti-manic agent is reasonable (Sit et al. 2018; Yatham et al. 2018). Receive treatment guidance and mood monitoring from a trained clinician. Light therapy is contraindicated for the patient with current or recent mania, hypomania or rapid cycling.
**Positioning and Selecting Your Light Box**. Figure 3.
Select a bright, white, UV-filtered light, 5,000 to 10,000 lux at a distance of 30–33 cm (12–13 inches) from the eyes. Utilise devices with an established record of ophthalmic safety (Gallin et al. 1995; Brouwer et al. 2017). The dimension of the ideal unit measures a minimum of 30 cm (12 inches) in height by 36 cm (14 inches) in length and is optimally placed on a desk stand to deliver illumination from above. The unit should produce diffuse (low-glare) lighting across a broad visual field. Avoid directly staring at the unit to mitigate eye discomfort but ensure light from device illuminates the visual field. Daily use of the light at the same time of day is strongly encouraged to gain the full benefit of treatment.
**Timing and Dosing Schedule**.
Begin with 15 min/day of light therapy. The session can be administered either in the morning (shortly after waking, between 7:00 AM and 9:00 AM) or at midday (between 12:00 PM and 2:30 PM), depending on patient preference and clinical recommendations. Increase by 15 min every week to a maximum of 45–60 min/day (by week 4) OR until mood symptoms have completely remitted and patient’s functioning is restored. Expect clinical remission by four to six weeks of treatment (Sit et al. 2018; Lam et al. 2021). For non-responders, move the timing of light to the morning within minutes after awakening (Sit et al. 2018; Sit and Haigh 2019). Morning light can provide a higher response compared to other times of day (Terman et al. 1998).
**Adverse Effects and Safety Monitoring**.
Headache, eye strain, agitation, nausea, sleep disturbance, and menstrual disturbances are potential side effects of light therapy. Suicidal ideation and manic switch are infrequent adverse effects of light therapy (Sit and Haigh 2019; Lam et al. 2021; Geoffroy et al. 2025). In addition to clinician monitoring, consider utilising self-reported measures to assess patient centred outcomes (Young et al. 1978; Sit et al. 2018; Sit and Haigh 2019; Pulido et al. 2025). No ocular disorders were observed in a five-year study (Gallin et al. 1995; Brouwer et al. 2017). In rare instances of worsening depression, hypomanic induction, suicidal ideation, or mood cycles in association with light treatment, patients are advised to reduce the light dose or stop using the light box for a few days and contact their clinician for proper guidance (Terman and Terman 2005; Geoffroy et al. 2025). On weekends, holidays or off hours, go to the nearest urgent care clinic, crisis unit or emergency room for immediate medical attention.
**Continuation of Treatment**.
We expect continued improvement after 6 weeks of treatment (Sit et al. 2018)(see dose response relationship). Given that patients with BD who stop maintenance treatment with antidepressants after one year have 2x the risk for relapse compared to patients who continue treatment (70% vs 36%), certain remitted patients may prefer to continue bright light therapy to prevent recurrence.
**Lifestyle Management**.
Sleep disturbance is associated with residual mood symptoms, recurrent depressive episode and suicide ideation (Esaki et al. 2021a). Young to mid-aged outpatients with BD (≤ 44 years old) are observed to have reduced sleep efficiency, prolonged latency to sleep-onset and increased duration of wake after sleep onset in association with evening white light illumination (Esaki et al. 2021a). Given this evidence, quantify the amount and sources of daily light exposure and explore ways to reduce the exposure to evening light. Reduced physical activity (Melo et al. 2016), delayed sleep phase (Takaesu et al. 2018) and later timing of circadian rhythm activity (Esaki et al. 2021a) are predictors of bipolar depressive episode relapse. In contrast, increased daytime light exposure (Esaki et al. 2019) and increased circadian rhythm activity with robust circadian amplitude are protective factors and associated with a reduction in bipolar depressive symptoms (Esaki et al. 2019) and decreased risk for bipolar mood relapse (Esaki et al. 2021a). To stay well and avoid recurrent depression, encourage patients to engage in regular exercise, meals, social contact, and outdoors activity, practice healthy sleep habits and avoid excessive delays in bedtime.
